# Learning Styles and Vocabulary Acquisition in Second Language: How the Brain Learns

**DOI:** 10.3389/fpsyg.2015.01800

**Published:** 2015-11-25

**Authors:** Manuela Macedonia

**Affiliations:** ^1^Information Engineering, Johannes Kepler Universität LinzLinz, Austria; ^2^Neural Mechanisms of Human Communication, Max Planck Institute for Cognitive and Brain SciencesLeipzig, Germany

**Keywords:** learning style, language learning, vocabulary acquisition, brain, sensorimotor learning, second language acquisition, embodiment and grounded cognition

In recent years, foreign language education has been focussing on learning styles. However, despite the quantity of articles and practice books, websites on the topic, and investment in teacher training, there is no empirical evidence for the existence of learning styles. Furthermore, if one agrees that it is the brain that learns, there should be indicators in the brain for the existence of learning styles, anatomically, and/or functionally. This is not the case. In this paper, the validity and reliability of tests assessing learning styles are questioned. Thereafter, following on basics of cognitive neuroscience and experimental evidence it is argued that the natural way for the brain to learn words is by collecting multiple sensory and sensorimotor experiences. In fact, evidence-based literature in the domain of vocabulary acquisition demonstrates that the inclusion of multiple modalities leads to best results. Impoverished linguistic input by allowing only one modality, for example only acoustic or visual input—the so called learning style (Pashler et al., 2008) of the student—reduces the chances of acquiring words. Also, the article briefly outlines brain related factors that lead to high performance in vocabulary learning.

A closer look at the literature on learning styles shows a multitude of models that classify learners in different types. Despite differences (for reviews see Pashler et al., 2008; Romanelli et al., 2009; Kaminska, 2014), learning style models have a common base: they sustain that not all individuals learn the same way. One of them, the VARK model (Fleming, 2001), categorizes learners as aural, visual, kinaesthetic, haptic, and learners who prefer to read and write. In second language instruction, this view implicates that a person classified as aural should learn vocabulary items by means of acoustic training, whereas a visual learner would optimize the learning outcome by reading and/or using flash cards that illustrate the word's semantics. However, despite being very popular, this position is not evidence-based. It is simply taken for granted with the intention to promote the learners' capacities. Also, it is not proven that assessments administered to determine the learning style to which a person belongs are valid and reliable. Nobody has proven that they measure what they claim. One major issue connected to these tests is that they are based on self-report. It has been suggested that in self-reports, subjects might lack introspection and that yes or no answers on personal experiences may not mirror reality (Paulhus and Vazire, 2007; Vazire and Solomon, 2015). Furthermore, there is an additional aspect to consider: tests on learning styles—supposed they were valid and reliable—might tell how a learner likes to acquire information on a conscious level. However, this does not automatically imply that preference leads to best learning outcome. In Western countries, L2 instructions make large use of listening activities, reading and writing exercises for vocabulary learning. These procedures are well-known to learners. When asked, learners may indicate them as preferences due to familiarity. Also, learners might not be informed about other possibilities of acquiring vocabulary and/or have not tested them. For example, learners might not know that performing gestures while learning words enhances retention compared with audio-visual learning (Macedonia, 2014). Hence, in order to define whether learners might acquire L2 words with one or another modality better, a large population should be tested in all modalities at different points in time. Testing should occur with vocabulary items that are controlled for familiarity, length, and associative features. If the population proves to repeatedly learn vocabulary items with visual input better than with aural or kinaesthetic and haptic input, then this population might have a learning style and the test would be valid. But such studies have yet to be conducted. Reviews on learning styles often come to the insight that best practice employs a variety of learning styles (Romanelli et al., 2009). However, despite the arguments above and the lack of a scientific basis, practitioners take learning styles seriously. In L2 lessons, teachers endeavor to offer “individualized” learning tools with the intention to augment learning outcomes in vocabulary acquisition.

Considering that it is the brain that learns, it is worth asking what happens at this level during word learning in L2. On average, if learners display no congenital or acquired neurological impairments, they should possess similar learning potentials. Billions of neurons process and store the incoming information in large networks. They include areas of the brain that deal with language (Friederici, 2011), cognitive control (Abutalebi, 2008), semantic processing (Binder et al., 2009; Binder and Desai, 2011) and multisensory integration (Seghier, 2012), memory (McClelland et al., 1995), and with stimulus specific regions as illustrated in Figure 1. At the processing level, if learners hear the German word *Himbeer* Engl. raspberry and read it, their auditory cortices will analyse and store the sounds (Dubois et al., 2013). Their left fusiform gyrus will process the letter sequence and memorize it (McCandliss et al., 2003). If the learners are additionally presented a real fruit, a multitude of stimuli will reach their brains. Smelling will engage the piriform cortex (González et al., 2006) and tasting, the anterior insula (Barros-Loscertales et al., 2012)—a gustatory area. Manipulating the raspberry and its pulp will create patterns of the texture in somatosensory regions (Sathian et al., 2011) and the motor cortex will store movement patterns necessary to grasp and hold the fruit (Hauk et al., 2004). Writing the word *Himbeere* and/or drawing the fruit will activate visual and motor regions (Yuan and Brown, 2015). Hence, the concept of the raspberry, and its German label *Himbeere* will be represented in the brain with large experience dependent sensorimotor networks (Pulvermüller and Fadiga, 2010).

**Figure 1:**
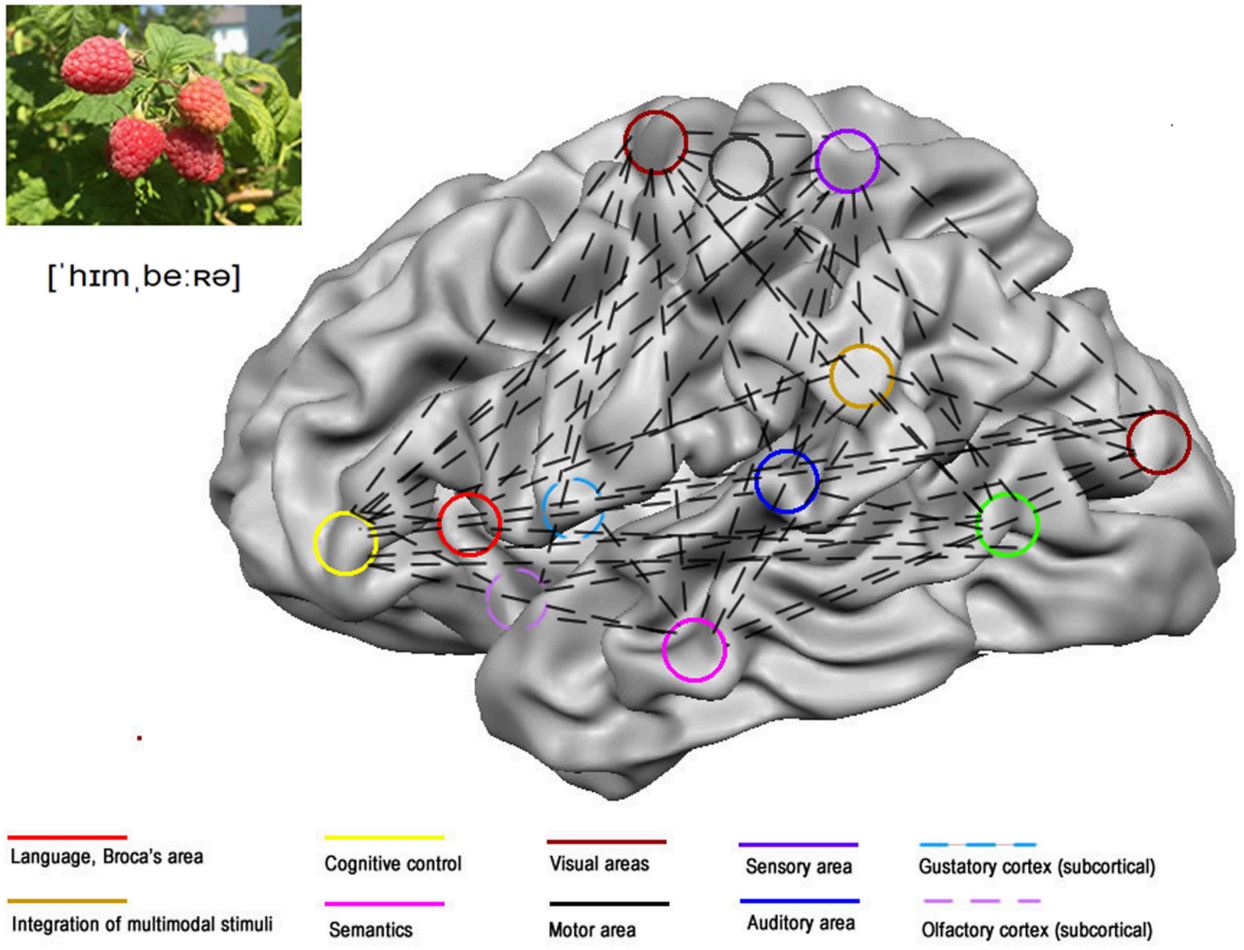
**Possible experience dependent word network for the German word *Himbeere***. Word networks reflect the experiences collected by the subject while learning. Natural learning engages a wide network comprising those regions in the brain that process the different aspects of the stimuli presented.

Having a particular learning style would imply that one region of the brain or network(s) selectively processes the information, i.e., more or better than another and therefore is in a way “dominant” over other regions. However, there is no scientific evidence that the brain does this. Whenever input is provided the brain processes and stores information in regions that are specifically engaged in this task. If synchronously active, brain regions wire together into functional networks (Hebb, 1949) sharing the information processed and stored. Hence, it is not surprising that smelling a rose without seeing it, allows one to imagine the color and shape of the flower (McClelland et al., 1986).

Most interesting for educators is the fact that the more complex brain networks are, the better they retain words (McClelland, 1985; Klimesch, 1994). A word network consisting of many components, i.e., visual, aural, kinetic, olfactory, etc. (Figure 1) stores and retrieves information more efficiently than a small network. If a component decays, for instance the sound sequence of the word, other components partially containing the lost information will restore it (Macedonia and Klimesch, 2014). This is due to the fact that all components of a network share and exchange information. Interestingly, behavioral memory research has been asserting for decades that word retention in L1 is impacted by the richness of stimuli accompanying the word (Craik and Tulving, 1975; Engelkamp and Zimmer, 1994). However, these findings have not reached L2 education and could not have an impact on procedures and methods.

In the early 1970s, behavioral research in L2 vocabulary learning worked on enrichment of vocabulary by means of pictures. Paivio and Csapo (1969) were among the first to recognize the power of pictures in L2 lessons. More recently, a number of empirical studies have confirmed that visual enrichment of words by means of pictures enhances memory (Curran and Doyle, 2011; Hockley and Bancroft, 2011; Bisson et al., 2014; Takashima et al., 2014). Similarly, if learners enrich L2 words with gestures, retention is enhanced in both the short and long term. Gestures engage a number of sensory modalities and the motor system, and thereby create very complex representations, i.e., word networks in the brain that highly impact retention: students learn more words and memorize words for longer than by only listening to the words and reading them (Macedonia et al., 2011; Macedonia, 2014). In a recent study, Mayer et al. (2015) compared the memory performance for words in L2 that had been learned according to three conditions: by reading only, by reading and enriching them through pictures, and by reading and performing semantically related gestures. Words that had only been read scored worst, whereas words learned with gestures scored best particularly in the long term. Results are no surprise: observing and thereafter self-performing a gesture requires more complex processing than observing a static picture. Altogether, empirical research on word learning demonstrates that enrichment of verbal information is key to word retention in L2 (Takashima et al., 2014) and to learning altogether (Shimojo and Shams, 2001; Seitz et al., 2006; Shams and Seitz, 2008; Thelen and Murray, 2013). Moreover, if one observes children while acquiring words in their L1, one will agree that they do not learn only acoustically or only visually. Instead, children collect multiple sensorimotor experiences related to words. Hence, it stands to reason that in L2 lessons “learning style specific” input, i.e., only acoustic or only visual, can by no means facilitate learning. Instead, it is possible that learning according to one's presumed learning style hinders learning.

If it is not a matter of learning styles matching with the teaching style of the instructor, why does performance vary so much among learners? Evidence-based research has found that subjects differ in their performance due to reasons related to their brain anatomy and function. Most interestingly, brain anatomy is not finally given at birth. Instead it changes with the use of our brains. A factor shaping brain anatomy that we encounter in classrooms is bilingualism. If a child grows up learning a second language, this induces structural changes in their white matter tracts, that is, the bilingual child's brain is wired better than that of a monolingual child (Li et al., 2014). Bilingualism also enhances cortical thickness in the inferior frontal gyrus: the core language region (Klein et al., 2014). Also, the age of L2 acquisition plays a major role: the younger the person is when they start learning L2, the larger the volume in sensory integration areas (Wei et al., 2015). In other words, in L2 classes, we find bilinguals and monolinguals and this can at least partially explain differences in learning performance. Also, phonological working memory, that is, the skill to keep unfamiliar sequences of sounds in mind, is also related to differences in word learning (Kapantzoglou et al., 2015). This has been recently demonstrated in a meta-analysis of 79 samples with around 3700 participants (Linck et al., 2014). Furthermore, two more mechanisms determine proficiency: first, cognitive control, the capacity to switch between L1 and L2 and to suppress interference from L1, a well investigated forebrain mechanism (Branzi et al., 2015), and second, attentional capacities (Bialystok, 2015). These factors that, for obvious reasons of space, here can only be mentioned are by no means connected to anything one could define as a learning style.

Learning style theories have not been scientifically demonstrated (Rogowsky et al., 2015), but many L2 teachers believe in them. Similarly, a multitude of L2-educators also believe that learners are right-brained or left-brained and try to improve their teaching to selectively activate the right hemisphere (Lindell, 2006; Lindell and Kidd, 2011). In both cases, we have to do with pseudoscience: It is appealing because simple, but unfortunate because as such it impacts education in a misleading way. In our time, we do have knowledge on learning processes and this knowledge should flow into L2 practice. Therefore, a basic education in cognitive neuroscience would prevent L2 teachers from becoming a soft target for pseudoscientific theories.

## Conflict of interest statement

The author declares that the research was conducted in the absence of any commercial or financial relationships that could be construed as a potential conflict of interest.
